# Neoadjuvant Chemotherapy in Patients With Muscle-Invasive Bladder Cancer and Its Impact on Surgical Morbidity and Oncological Outcomes: A Real-World Experience

**DOI:** 10.3389/fsurg.2018.00058

**Published:** 2018-09-19

**Authors:** Thanh-Tuan Nguyen, Olivier Huillard, Yohann Dabi, Julien Anract, Mathilde Sibony, Marc Zerbib, Evanguelos Xylinas

**Affiliations:** ^1^Department of Urology and Oncology, Cochin Hospital, Assistance Publique-Hôpitaux de Paris, Paris Descartes University, Paris, France; ^2^Urology Department, University of Medicine and Pharmacy at Ho Chi Minh City, Cho Ray Hospital, Ho Chi Minh City, Vietnam

**Keywords:** bladder cancer, cystectomy, morbidity, neoadjuvant chemotherapy, survival

## Abstract

**Objectives:** The purpose of this study was to investigate the impact of neoadjuvant chemotherapy (NAC) on perioperative morbidity and on oncological outcomes according to the type of chemotherapy regimen administered to patients with muscle-invasive bladder cancer (MIBC) who subsequently underwent radical cystectomy (RC).

**Methods:** Data were collected retrospectively on 40 patients with bladder urothelial carcinoma who had at least two cycles of NAC, followed by RC, from 2011 to 2015 at our institution. The outcomes evaluated were NAC toxicity, perioperative complications, cancer-specific, and overall survival.

**Results:** Among these cases, 23 patients (57.5%) received methotrexate, vinblastine, doxorubicin and cisplatin (MVAC), 4 patients (10%) received gemcitabine and cisplatin (GC), and 13 patients (32.5%) received other regimes. The early and late postoperative complication rates were 35% and 12.5%. Regarding toxicity, 85% of patients had at least one side effect of NAC, but only 21.7% discontinued therapy in the MVAC group. The pathological complete response (pCR) rates for cisplatin-based regimens (MVAC and GC) and other regimens were 44.4 and 15.4%, respectively, (*p* = 0.09). The pathological partial response (pPR) rates for cisplatin-based regimens and other regimens were 66.7 and 15.4%, respectively, (*p* = 0.002). Patients treated with a cisplatin-based chemotherapy regimen had longer overall survival than those treated with other regimen (median 38.1 vs. 18.4 months, *p* = 0.01).

**Conclusions:** NAC administration was not associated with high toxicity or surgical morbidity. The pathological response rates and survival outcomes in the cisplatin-based regimens were higher than with those with non-cisplatin-based regimens. These data support the use, in patients elective to a neoadjuvant setting prior to RC for MBIC, of a cisplatin-based regimen.

## Introduction

Muscle-invasive bladder cancer (MIBC) is an aggressive disease with a high-risk of early metastasis and cancer-specific mortality. The gold standard treatment of MIBC is radical cystectomy (RC) in conjunction with concomitant bilateral pelvic lymphadenectomy (1). The prognosis of these patients depends mainly on the histologically determined pathological stage of the radical cystectomy specimen (pT stage) and on the presence of lymph node metastases (pN status). Without administration of chemotherapy, 90.5% of MIBC patients undergoing radical cystectomy have a 10-year cancer-specific free survival (CSS) if the pT stage is pT0/a/is/1 pN0 (non-residual disease or non-muscle invasive residual disease). The 10-year CSS decreases to 67% if this stage is pT2 pN0 (muscle invasive confined disease). Only 16.7% of patients with nodal metastasis at the time of RC have 10-year cancer-specific free survival, independent of the primary tumor stage (2).

Due to the development and implementation of neoadjuvant chemotherapy (NAC) prior to radical cystectomy, the prognosis for MIBC patients undergoing radical cystectomy has improved (3, 4). The goals of neoadjuvant chemotherapy administration are to (1) eradicate the micrometastases, (2) avoid the release and implantation of malignant cells during cystectomy, and (3) extend the survival of these patients.

In the Southwest Oncology Group (SWOG) Intergroup study, RC alone was compared with three cycles of neoadjuvant MVAC (methotrexate, vinblastine, doxorubicin, and cisplatin), followed by radical cystectomy (3). This randomized trial showed that the median survival duration was 77 months in patients with combination treatment, and 46 months in patients with upfront RC alone (3). In terms of overall survival (OS), the group combination therapy recipients had better outcomes (5-year OS = 57 vs. 43%, *p* < 0.05) (3). In addition, the rate of stage pT0 disease in the radical cystectomy specimens was significantly higher among the patients who received MVAC combination (3). In the International Collaboration of Trialists study, 976 patients with tumor stage cT2-4N0 were randomized into two groups and underwent either three cycles of neoadjuvant cisplatin, methotrexate, vinblastine (CMV), or no neoadjuvant therapy following local therapy alone. Local therapy comprised either radiotherapy, radical cystectomy, or a combination of low-dose radiotherapy and radical cystectomy (5). This randomized prospective trial showed that CMV combination allowed a 16% reduction in the risk of death. Following NAC, pathological complete response was 32.5 vs. 12.3% with radical cystectomy alone (5). Based on these data, cisplatin-based neoadjuvant chemotherapy has demonstrated an improvement in OS in MIBC and has reached a grade recommendation (1, 3–5).

However, NAC is rarely administrated in daily practice. In the United States, an analysis of the National Cancer Data Base showed that only 16% of patients with MIBC underwent NAC (6). In Western and Central Europe, the rate of NAC administration was also low, with only 12% of patients with MIBC undergoing cystectomy having received NAC (7). The reasons for this low implementation rate of NAC include, among general toxicity concerns, increased perioperative complications, potentially ineffective chemotherapy, and delay in curative-intent surgery. Therefore, the management of MIBC remains controversial, with debate existing over neoadjuvant chemotherapy plus radical cystectomy, vs. upfront surgery alone.

Therefore, the present study retrospectively analyzed the impact on surgical morbidity and on the oncological outcomes of MIBC patients at our institution who underwent radical cystectomy with prior neoadjuvant chemotherapy.

## Methods

### Patients

The study was designed as a retrospective investigation, in which 50 consecutive patients treated with neoadjuvant chemotherapy before undergoing radical cystectomy with bilateral pelvic lymphadenectomy at Cochin hospital from July 2011 to July 2015 were included. Patients who had indication of radical cystectomy (cT2-4aNxM0 or high risk T1) and received at least two cycles of NAC followed by RC were selected for further analysis. Patients with any other histological type that pure urothelial carcinoma or mixed histology with squamous and/or glandular differentiation were excluded. Our final cohort included 40 patients [10 were excluded because of < 2 cycles of chemotherapy (*n* = 8) and because of lost to follow-up (*n* = 2)].

### Pathological analyses

All transurethral resection and cystectomy specimens underwent pathological review by specialist genitourinary pathologists at our institution. Clinical staging was based on transurethral resection of bladder tumor (TURBT) and preoperative imaging. Postoperative pathological analyses included tumor classification, presence of carcinoma *in situ*, surgical margin status, number of lymph nodes and TNM staging according to the 2010 American Joint Committee on cancer classification. One of our primary outcomes was pathological response to neoadjuvant chemotherapy. Complete pathological response was defined as pT0N0 stage on the radical cystectomy specimen and partial pathological response was defined as pT0N0, pTaN0, pTisN0, or pT1N0.

### Therapeutic sequence

All patients were included in a multidisciplinary tumor board at which NAC administration was decided. The choice of chemotherapy regimen was then made at the referent oncologist's discretion based on patient's clinical features. The NAC regimens included MVAC, GC (gemcitabine and cisplatin) or Other. Typical regimen of MVAC chemotherapy was four cycles at 28-day intervals over 16 weeks with methotrexate 30 mg/m^2^ (days 1, 15, and 22), vinblastine 3 mg/m^2^ (days 2, 15, and 22), doxorubicin 30 mg/m^2^ (day 2), and cisplatin 70 mg/m^2^ (day 2). Patients in the GC group received typically four cycles at 21-day interval over 12 weeks with gemcitabine 1,000 mg/m^2^ (days 1, 8, and 15) and cisplatin 70 mg/m^2^ (day 2). The Other group consisted of patients receiving gemcitabine with carboplatin, GON (gemcitabine, oxaliplatine, and navelbine) or GEMOX (gemcitabine and oxaliplatin). During NAC administration, the dose of chemotherapy was adjusted based on creatinine and chemotherapy toxicities. NAC data included types of regimen, number of cycles, change of NAC dose, and toxicities of chemotherapy.

Following neoadjuvant chemotherapy, radical cystectomy with standard pelvic lymphadenectomy was performed on all patients. The operative variables included the time between diagnosis and surgery, type of urinary diversion and surgical morbidity. During the follow-up, we analyzed the perioperative complications of RC according to the Clavien classification system. Early and late complications were defined as a post-operative complication within 30 days and from 30 to 90 days, respectively.

After surgery, patients were seen every 3 months during the first year, every 6 months the year after and every year after that. Radiological exams (CT abdomen /pelvis with intravenous contrast) were practiced every year or at any moment if the physician suspected disease recurrence. Disease recurrence was defined as tumor relapse in the operative field, regional lymph nodes and/or distant metastases. Death's cause was determined using patient's death certificate. Death within 30 days after surgery was censored for specific survival analysis.

The oncological outcomes included pathological response, OS and CSS.

### Statistical analyses

Variables collected in the database included patient demographics, clinical staging, NAC regimen features, radical cystectomy procedure data, pathological features, surgical morbidity, postoperative morbidity, and oncological outcomes. For variables with non-normal distribution, data were presented as median and interquartile range (IQR). Means and medians were reported for continuous variables. Proportions and frequencies were reported for categorical variables. Means were compared by using the Student's T-test. For variables with non-normal distribution, the two groups were compared by using the Mann-Whitney U test. When there were more than three groups, the Kruskal–Wallis test was used. Proportions were compared by using Chi-squared tests with continuity correction of Fisher's exact test when appropriate. The Kaplan-Meier method and multivariable Cox proportional hazards regression model were used to analyze occurrence of cancer-specific and overall mortality. The analyses were performed with the SPSS software version 20.0 (IBM SPSS Statistics; IBM Corp, Armonk, NY, USA).

## Results

### Patient demographics and clinical features

The patient characteristics, the surgical features and pathological outcomes according to the type of NAC are shown in Table 1. The median age of the population was 65 (IQR, 61–71 years) and the ratio of males to females was 4:1. In the Other regimens group, eight patients received GEMOX, four received GON and one patient received carboplatin with gemcitabine. There were no differences between the different NAC groups with regards to gender, clinical stage of tumor at diagnosis, associated CIS, histological grade of tumor, or hydronephrosis at presentation and time between last TURBT and cystectomy. However, there was a significant difference in age among the three groups (*p* = 0.044).

**Table 1:** Clinical characteristics, operative features, and pathologic outcomes of patients receiving neoadjuvant chemotherapy.

**NAC regimen**	**MVAC (*n* = 23)**	**GC (*n* = 4)**	**Other (*n* = 13)**	**Total**
Age at RC, year, median (IQR)	62 (56–70)	70 (65–74)	68 (65–75)	65 (61–71)
Male, *n* (%)	20 (87)	3 (75)	9 (69.2)	32 (80)
**CLINICAL STAGE AT PRESENTATION**, ***n*** **(%)**				
T1	2 (8.7)	0	1 (7.7)	3 (7.5)
T2	20 (87)	3 (75)	8 (61.5)	31 (77.5)
T3	1 (4.3)	1 (25)	2 (15.4)	4 (10)
T4a	0	0	2 (15.4)	2 (5)
N0	17 (73.9)	4 (100)	6 (46.2)	27 (67.5)
N1	2 (8.7)	0	1 (7.7)	3 (7.5)
N2	4 (17.4)	0	6 (46.2)	10 (25)
Associated CIS, *n* (%)	3 (13)	0	0	3 (7.5)
**HISTOLOGIC GRADE OF TUMOR**				
2	1 (4.3)	0	0	1 (2.5)
3	22 (95.7)	4 (100)	13 (100)	39 (97.5)
**PRESENCE OF HYDRONEPHROSIS**, ***n*** **(%)**				
No hydronephrosis	19 (82.6)	3 (75)	6 (46.2)	28 (70)
Unilateral hydronephrosis	3 (13)	1 (25)	6 (46.2)	10 (25)
Bilateral hydronephrosis	1 (4.3)	0	1 (7.7)	2 (5)
Median of the time between last TURBT and cystectomy, days, (IQR)	133 (111–154)	161 (136–265)	156 (106–176)	142.5 (112–163)
**NUMBER OF CYCLES OF NAC COMPLETED**, ***n*** **(%)**				
2	2 (8.7)	1 (25)	0	3 (7.5)
3	3 (13)	1 (25)	1 (7.7)	5 (12.5)
4	11 (47.8)	2 (50)	9 (69.2)	22 (55)
≥5	7 (30.4)	0	3 (23.1)	10 (25)
Change of NAC dose or regime during chemotherapy, *n* (%)	3 (13)	2 (50)	2 (15.4)	7 (17.5)
**TYPE OF URINARY DIVERSION**, ***n*** **(%)**				
Ileal conduit	9 (39.1)	2 (50)	10 (76.9)	21 (52.5)
Orthotopic neobladder	14 (60.9)	2 (50)	3 (23.1)	19 (47.5)
**PATHOLOGIC OUTCOME**				
pT0N0	12 (52.2)	0	2 (15.4)	14 (35)
≤ pT1N0	17 (73.9)	1 (25)	2 (15.4)	20 (50)
Positives nodes, median (range)	0 (0-1)	0.5 (0-1)	0 (0-3)	0 (0-3)
Positive surgical margins, *n* (%)	1 (4.3)	0	2 (15.4)	3 (7.5)
Associated CIS post-operation, *n* (%)	4 (17.4)	0	2 (15.4)	6 (15)

### Neoadjuvant chemotherapy

NAC toxicities are displayed in Table 2. In this study, 85% of patients suffered at least one adverse effect. The proportions of incomplete chemotherapy due to toxicity (less than four cycles of NAC) were 21.7% for MVAC, 50% for GC, and 7.7% for the Other regimens (7.7%) (*p* = 0.172).

**Table 2:** Toxicity of neoadjuvant chemotherapy.

**Toxicity, *n* (%)**	**Grade 1**	**Grade 2**	**Grade 3**	**Grade 4**
Neutropenia	3 (7.5)	3 (7.5)	2 (5)	1 (2.5)
Anemia	5 (12.5)	1 (2.5)	1 (2.5)	0
Thrombocytopenia	6 (15)	1 (2.5)	2 (5)	0
Gastrointestinal effects (nausea, vomiting, diarrhea, or constipation)	12 (30)	5 (12.5)	2 (5.0)	0
Renal insufficiency	5 (12.5)	0	0	0
Mucositis	3 (7.5)	2 (5)	0	
Neuropathy	4 (10)	1 (2.5)	0	0
Fatigue or malaise	9 (22.5)	3 (7.5)	1 (2.5)	0
Infection	2 (5)	1 (2.5)	0	0

### Surgical morbidity

Early and late morbidity data for patients treated with NAC prior to RC are presented in Table 3. This study showed that 35% of patients had at least one early complication after RC followed by one late complication in 12.5% cases. Overall minor (grade 1-2) and major (grade 3-5) complications rates were 66.7 and 33.3%, respectively. In the group of late complication, two patients died secondly to an intestinal obstruction and to a septicemia.

**Table 3:** Early and late Postoperative complications after neoadjuvant chemotherapy and radical cystectomy.

**Complication, *n* (%)**	**Early complication (** ≤ **30 days)**					**Late complication (**>**30 days and** ≤ **90 days)**				
	**Grade 1**	**Grade 2**	**Grade 3**	**Grade 4**	**Grade 5**	**Grade 1**	**Grade 2**	**Grade 3**	**Grade 4**	**Grade 5**
Wound infection	0	0	0	0	0	0	0	0	0	0
Urinary infection	0	8 (20)	1 (2.5)	1 (2.5)	0	0	1 (2.5)	0	0	1 (2.5)
Hemorrhage	0	0	0	1 (2.5)	0	0	0	0	0	1 (2.5)
Urinary leakage	0	1 (2.5)	0	0	0	0	0	0	0	0
Ileus	0	1 (2.5)	1 (2.5)	0	0	0	0	0	0	0
Renal effects (acute renal insufficiency)	0	3 (7.5)	0	0	0	0	0	2 (5)	0	0
Lymphoceles	1 (2.5)	0	0	0	0	0	0	0	0	0
Thrombosis or embolism (pulmonary embolism, deep venous thrombosis)	0	1 (2.5)	0	0	0	0	0	0	0	0
Total	1 (2.5)	14 (35)	2 (5)	2 (5)	0	0	1 (2.5)	2 (5)	0	2 (5)

### Pathological outcomes

The pathological outcomes stratified by the NAC regimens are displayed in Table 4. The pCR rates were significantly higher (44.4%) for the cisplatin-based regimens (MVAC and GC) vs. 15.4% for the other regimens (*p* = 0.09). The pPR rates for the cisplatin-based regimens and other regimens were 66.7 and 15.4%, respectively, (*p* = 0.002).

**Table 4:** Correlation of clinical stage and final pathologic stage stratified by NAC.

	**pT0N0 (pCR)**	**pTis/pTa/pT1N0**	**≤ pT1N0 (pPR)**	**pT2N0**	**pT3–4N0**	**pTanyN1-3**	**Total**
**MVAC NAC**, ***n*** **(%)**							
cT1	0	1 (50)	1 (50)	0	1 (50)	0	2
cT2	12 (60)	3 (15)	15 (75)	1 (5)	3 (15)	1 (5)	20
cT3	0	1 (100)	1 (100)	0	0	0	1
cT4	0	0	0	0	0	0	0
Total	12 (52.2)	5 (21.7)	17 (73.9)	1 (4.3)	4 (17.4)	1 (4.3)	23
**GC NAC**, ***n*** **(%)**							
cT1	0	0	0	0	0	0	0
cT2	0	1 (33.3)	1 (33.3)	0	1 (33.3)	1 (33.3)	3
cT3	0	0	0	0	0	1 (100)	1
cT4	0	0	0	0	0	0	0
Total	0	1 (25)	1 (25)	0	1 (25)	2 (50)	4
**OTHER NAC**, ***n*** **(%)**							
cT1	0	0	0	0	0	1 (100)	1
cT2	2 (25)	0	2 (25)	1 (12.5)	3 (37.5)	2 (25)	8
cT3	0	0	0	0	2 (100)	0	2
cT4	0	0	0	0	0	2 (100)	2
Total	2 (15.4)	0	2 (15.4)	1 (7.7)	5 (38.5)	5 (38.5)	13

*GC, gemcitabine and cisplatin; MVAC, methotrexate, vinblastine, doxorubicin, cisplatin; NAC, neoadjuvant chemotherapy; pCR, pathologic complete response; pPR, pathologic partial response*.

### Survival outcomes

**Figure 1:**
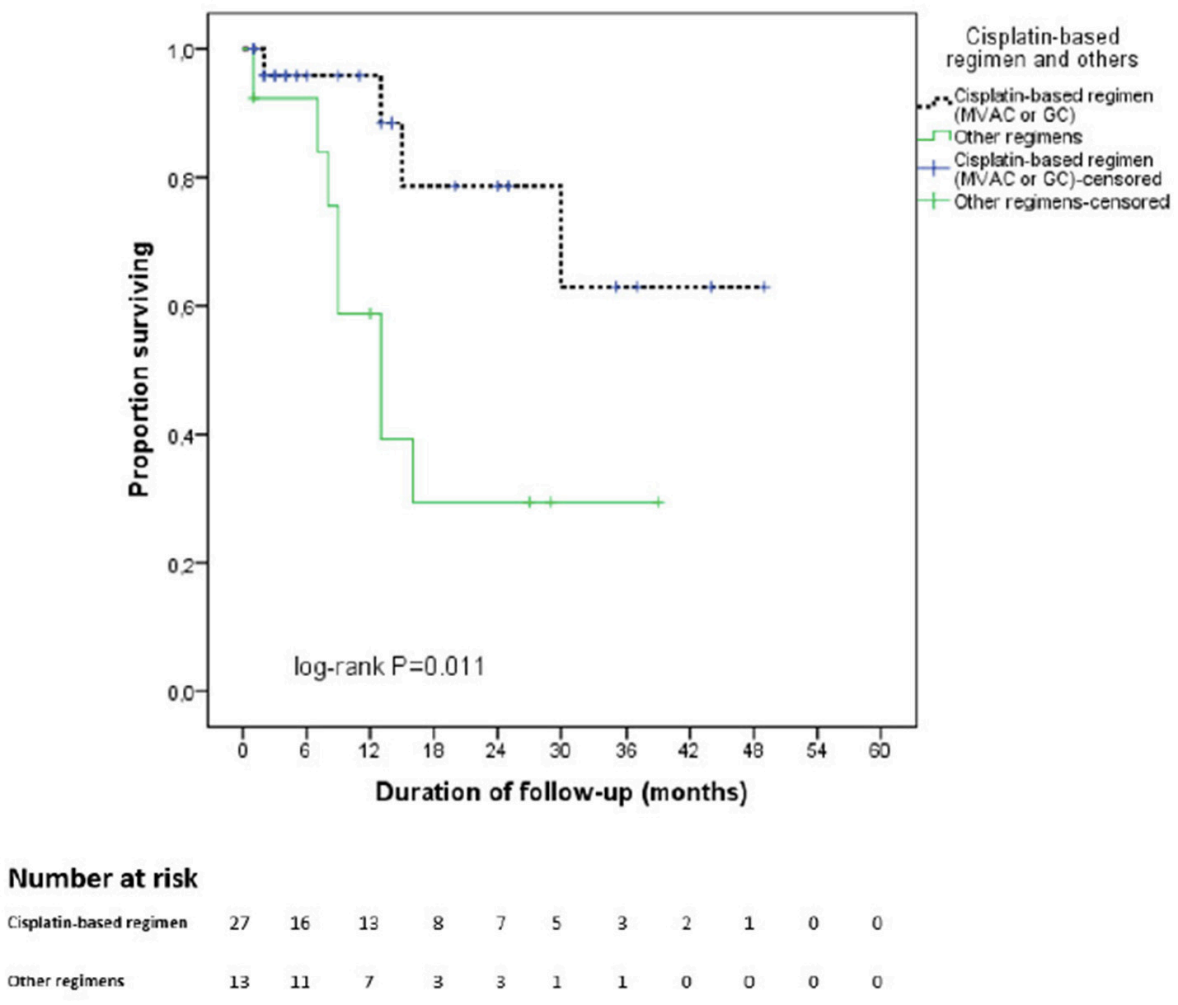
Kaplan Meier curve for overall survival stratified by type of neoadjuvant chemotherapy regimen. In black: cisplatin—based chemotherapy regimen. In green: other regimens. cnoinall survival elected in a t benefitsing prior to RC for MBIC ts with lower performance status that were not selected in at cn.

In the entire cohort, the median duration of follow-up was 21.5 months and the estimated mean OS time was 30.7 months. Figure 1 displays the Kaplan-Meier curves for OS stratified by NAC regimen. Specifically, patients were divided into cisplatin-based regimens (MVAC or GC) and Other regimens. Patients receiving cisplatin-based regimens had better OS outcomes (38.1 vs. 18.4 months, *p* = 0.011).

**Figure 2:**
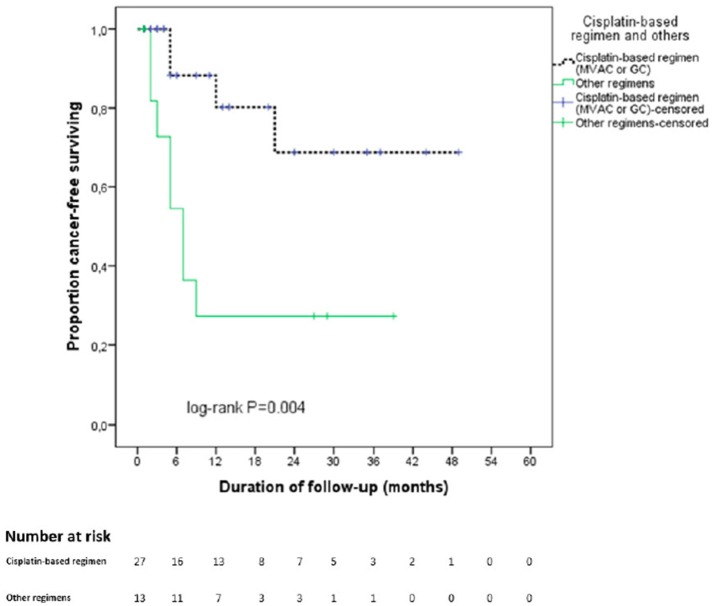
Kaplan Meier curve for cancer specific survival stratified by type of neoadjuvant chemotherapy regimen. In black: cisplatin—based chemotherapy regimen. In green: other regimens.

The estimated CSS mean time for the entire cohort was 30.1 months based on Kaplan-Meier method. Figure 2 displays the Kaplan-Meier curves for CSS stratified by NAC regimens. Patients receiving cisplatin-based regimens had better CSS outcomes (37.6 vs. 14.3 months, *p* = 0.004).

**Figure 3:**
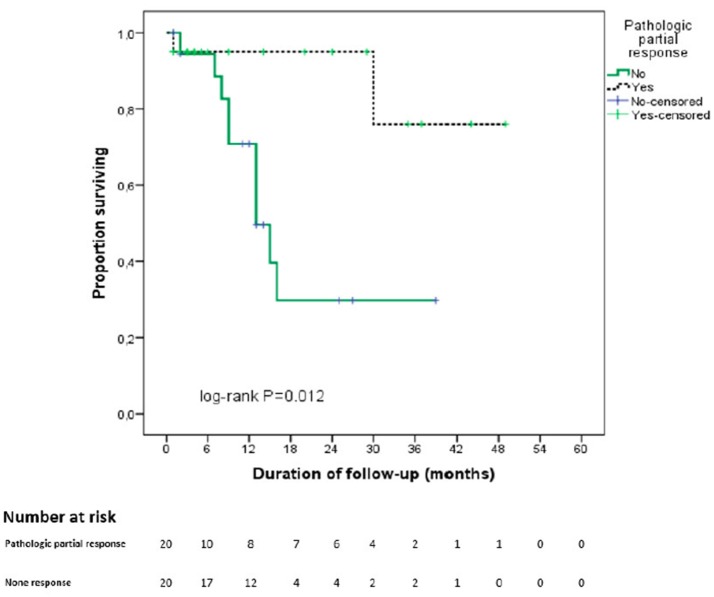
Kaplan Meier curve for overall survival stratified by pathological partial response. In black: partial pathological response on final pathological analysis. In green: absence of partial pathological response on final pathological analysis.

With regards to pathological response, Figure 3 shows the Kaplan-Meier curves for OS of the two groups with and without pathological partial response. The OS of patients with pPR was better than that of the remaining patients (43 vs. 19.5 months, *p* = 0.012).

In the Cox proportional hazards regression model for overall mortality, age (HR: 1.17 [95% CI, 1.02-1.33]) and positive surgical margins status (HR: 11.93 [95% CI, 1.13–125.81]) were significantly associated with overall mortality (Table 5).

**Table 5:** Cox regression model assessing factors predicting overall mortality occurrence.

**Variable**	**Cox regression analysis**
	**HR (95% CI)**	***p*****-value**
Age	1.17 (1.02–1.33)	**0.024**
**GENDER**		
Male	1	
Female	1.62 (0.27–9.81)	0.6
**CHEMOTHERAPY REGIMEN**		
MVAC	1	
GC	3.23 (0.22-48.45)	0.4
Other regimens	3.05 (0.28-32.95)	0.36
Surgical margin
Negative	1	
Positive	11.93 (1.13–125.81)	**0.04**
**PCR (PT0N0)**		
No	1	
Yes	0.61 (0.07–5.07)	0.65

*P-value < 0.05 is significant which means age and positive surgical margins status were significantly associated with overall mortality*.

## Discussion

One disinclination to the wider administration of NAC derives from the concern of increased postoperative complications after radical cystectomy in patients receiving this regimen. The results of our study showed that morbidity and mortality during early and late period of follow-up (30 and 90 days, respectively) were similar to the reported complications rates of upfront radical cystectomy series (without administration of NAC) (2, 8–12). Previous studies reported that the early complications rates were 19.7–39.5%, the late complications rates were 10.8–19.8%, the early mortality rates were 0.4–3.2% and finally the late mortality rates were 3 to 9% (2, 8–12). Our results are in line with those of Johnson et al. (13) who showed no increased perioperative complications/surgical morbidity when comparing two groups of patients treated with RC, one with NAC administration and one without (13). Therefore, NAC is not associated with excessive post-operative morbidity and should be recommended as long as it improves the oncological outcomes.

Several studies emphasized that MVAC-based NAC administration improves survival in patients treated with RC (3, 5, 14–16). Moreover, several studies revealed that GC regimens was associated with similar pathological response rates and survival outcomes compared to MVAC regimens (4, 17–19). In this study, we showed that patients receiving cisplatin-based regimens, the pCR and pPR were 44.4 and 66.7%, respectively. These data were higher than previous reported studies where the pCR following NAC varied from 22.7 to 38% (3, 5, 20). This difference could be explained by the low proportion of locally-advanced disease before NAC in our cohort compared to previous reported cohorts. In fact, the proportion of cT3-4a in the cisplatin-based group of our series (7.4%) was lower for example than in the MVAC group of SWOG trial and the Nordic Urothelial Cancer Group trial (48.6 and 59.1%) (3, 20). In our cohort, similar to a multicenter study published by Zargar et al. (4), the pPR rate of the cisplatin-based regimen was superior significantly to the other regimen group (66.7 vs. 15.4%, *p* = 0.002) (4). The pCR rates of the cisplatin-based regimens were better than those of the other regimens, although the difference was not statistically significant (44.4 vs. 15.4%, *p* = 0.09). The pathological response after NAC is strongly associated with overall survival and is one of the most important factors in order to evaluate the efficacy of NAC (4, 20, 21). Similarly, we found out that OS of the pPR group was higher compared to the rest of our cohort (43 vs. 19.5 months, *p* = 0.012). The OS was as well higher in the pCR group compared to the rest of our series, although the difference was not statically significant (39.7 vs. 26 months, *p* = 0.26). These rates are lower than those reported by Grossman et al. in the SWOG study. One possible explanation is that we included patients with lower performance status representing perhaps a more real-life cohort than the patients usually selected in a clinical trial. Previous studies have reported that the patients harboring a pT0 stage at RC had a survival benefit (22, 23). In the NAC setting, the use of cisplatin-based regimen was a predictor of favorable pathological response which might translates into survival benefits.

Our study harbors important limitations mainly due to its retrospective design. First, the choice of NAC regimen was influenced by many factors such as performance status, renal function, and comorbidity of patients that could also affect the outcomes. Therefore, our series have some imbalances in patient characteristics between the NAC regimen groups. Secondly, our study was also limited by a small sample size that challenges the statistical analyses. Third, we did not assess the information on NAC dose density, growth factor support and performance status. These limitations may impact our estimates of pathological response and survival outcomes. Nonetheless, we could determine the perioperative morbidity and oncological outcomes according NAC regimens. One strength of our study is that we report a real-world practice cohort different from the selected patients usually enrolled in the clinical trials.

In conclusion, our results augment the body of evidence of the previously reported beneficial effects of NAC from a daily practice viewpoint. NAC administration was not associated with high toxicity or surgical morbidity. The pathological response rates and survival outcomes in the cisplatin-based regimen groups were higher than those in the non-cisplatin-based regimen groups. These data support the use of the cisplatin-based regimen in the neoadjuvant setting prior to RC for MIBC.

## Ethics statement

This study was carried out in accordance with the recommendations of French guidelines, CPP committee with written informed consent from all subjects.

All subjects gave written informed consent in accordance with the Declaration of Helsinki. The protocol was approved by the CPP from Ile de France.

## Author contributions

All authors listed have made substantial, direct, and intellectual contribution to the work and approved it for publication.

### Conflict of interest statement

The authors declare that the research was conducted in the absence of any commercial or financial relationships that could be construed as a potential conflict of interest.

## Abbreviations

CFS: cancer-free survival
DSS: disease specific survival
GC: gemcitabine and cisplatin
GON, gemcitabine, oxaliplatine: and navelbine
GEMOX: gemcitabine and oxaliplatin
HR: hazard ratios
IQR: interquartile range
MIBC: Muscle-invasive bladder cancer
MVAC, methotrexate, vinblastine: doxorubicin and cisplatin
ACG, nab-paclitaxel: carboplatin and gemcitabine
NAC: neoadjuvant chemotherapy
OS: overall survival
pCR: pathological complete response
pPR: pathological partial response
RC: radical cystectomy
SWOG: Southwest Oncology Group
TURBT: transurethral resection of bladder tumor.
